# Hole-Transporting Materials Based on a Fluorene Unit for Efficient Optoelectronic Devices

**DOI:** 10.3390/ma17225417

**Published:** 2024-11-06

**Authors:** Maoli Man, Mingming Zhao, Yunfei Lyu

**Affiliations:** 1 Hebei Petroleum University of Technology, Chengde 067000, China; manmaoli@163.com; 2 School of Chemical Engineering and Technology, Tianjin University, Tianjin 300354, China; 3014207421@tju.edu.cn

**Keywords:** solution-processable materials, hole-transporting materials, carrier mobility, OLED, long-afterglow materials

## Abstract

Solution-processable hole-transporting materials (HTMs) that form highly soluble films and thermally stable amorphous states are essential for advancing optoelectronic devices. However, the currently commercialized HTM, N,N-bis(3-methylphenyl)-N,N0-bis(phenyl)benzidine (TPD), exhibits poor solubility and limited carrier transport when spin-coated into thin films. Herein, to address these issues, a fluorenyl group was ingeniously incorporated into a series of molecules structurally similar to TPD. The resulting compounds, namely, 2,7-di-(N,N-diphenylamino)-9,9-dimethyl-9H-fluorene (DDF), 2,7-di-p-tolyl-(N,N-diphenylamino)-9,9-dimethyl-9H-fluorene (2M-DDF), and 2,7-di-tetra-p-tolyl-(N,N-diphenylamino)-9,9-dimethyl-9H-fluorene (4M-DDF), offered tunable energy levels, carrier transport, crystallinity, and steric configuration via adjustment of the number of terminal methyl groups. Owing to its satisfactory performance, 2M-DDF can serve as an effective alternative to TPD in OLED devices as well as a guest molecule in host–guest systems for long-afterglow materials. Devices incorporating 2M-DDF as the HTM, with an Alq_3_ emitter, achieved a maximum CE of 4.78 cd/A and a maximum *L* (*L*_max_) of 21,412 cd m^−2^, with a turn-on voltage (*V*_on_) of 3.8 V. The luminous efficiency of 2M-DDF was approximately five times that of TPD (4106 cd m^−2^). Furthermore, when 2M-DDF and TPD were utilized as guest molecules in afterglow materials, the afterglow duration of 2M-DDF (10 s) was 2.5 times that of TPD (4 s). This study provides a theoretical basis for the development of high-performance HTMs and long-afterglow materials, establishing a framework for the application of fluorene-based compounds in emerging fields such as long-afterglow materials.

## 1. Introduction

Efficient organic optoelectronic devices such as solar cells, large-area displays, long-afterglow materials [1,2,3,4], phosphorescent room-temperature materials [5,6,7,8,9], organic light-emitting diodes (OLEDs) [10,11,12,13,14], and organic perovskite solar cells [15,16,17,18,19] rely on organic materials with high charge transport ability. OLEDs and phosphorescent materials with a long afterglow at room temperature are especially meritorious [20,21] for portable display devices and can even realize large-area display panels. However, the difficulty of designing and synthesizing thermally robust emissive materials with high hole-transport mobility and improved multi-functional properties has limited the development of next-generation high-performance optoelectronic devices [22,23,24,25,26]. Some widely used and relatively inexpensive hole-transporting materials [27,28,29,30,31] such as triphenylamine, carbazole (CA), and their derivatives demonstrate excellent hole mobility and high chemical stability. The common HTMs are N,N-diphenyl-N,N-bis(1-naphthyl)-(1,10-biphenyl)-4,40-diamine (NPB), and TPD, which possess high charge-carrier mobility and are easily sublimated [32,33,34]. However, the low solubility of these materials limits their large-scale application [35,36].

Fluorene-based conjugated compounds exhibit significantly enhanced solubility due to the presence of methyl groups linked to the 9-position of fluorine, this is mainly because the alkyl chains have the ability to hinder intermolecular packing [37], along with a high energy band gap (HOMO: −5.5~−5.2 eV, LUMO: −2.6~−2.0 eV) and superior electron and charge double transport capacity (10^−4^~10^−5^ cm^2^V^−1^S^−1^), which has garnered increasing attention from scholars [38,39]. For instance, Okumoto and Shiroto et al. [39] employed the Suzuki coupling method to synthesize a symmetrical fluorene compound with biphenyl as the core, which serves as a hole-transport material exhibiting high hole mobility and excellent thermal stability. However, due to the compound’s symmetrical structure, it tends to form a regular and tightly packed arrangement during film formation or device utilization, leading to crystallization issues that ultimately compromise device performance, efficiency, and lifespan. Consequently, the practical application of this material is limited.

Inspired by the structure of TPD and combined with the advantages of fluorene-based compounds, the DDF compound was synthesized. We further introduced a methyl group through the end group to control the crystallization performance, so 2,7-di-p-tolyl-(N,N-diphenylamino)-9,9-dimethyl-9H-fluorene (2M-DDF), and 2,7-di-tetra-p-tolyl-(N,N-diphenylamino)-9,9-dimethyl-9H-fluorene (4M-DDF) were synthesized in this study. The thermal, electrochemical properties and carrier mobility of the three compounds were investigated through thermogravimetric analysis (TGA), differential scanning calorimetry (DSC), cyclic voltammetry (CV), and space charge limited current (SCLC). The DDF, 2M-DDF, and 4M-DDF compounds have good solubility, thermal stability, and are easily fabricated as thin films by spin coating. More advantageous is the high carrier mobility in the hole-transport layers of the 2M-DDF prepared via spin coating. Consequently, an OLED device with 2M-DDF as the hole-transport layer and Alq_3_ as the emitter achieved superior performance, with a maximum current efficiency (*CE*) of 4.78 cd/A, a *V*_on_ of 3.80 V, and a luminance (*L*) of 21,412 cd/m^2^. Compared with traditional OLED hole-transport materials TPD and poly[(9,9-dioctylfluorenyl-2,7-diyl)-alt-(4,4′-(N-(4-butylphenyl) (TFB) materials with similar structure, the maximum luminous brightness is increased by nearly five times (4106 cd m^−2^) and 1.3 times(15,211 cd m^−2^), respectively, and the luminous performance is greatly improved, indicating that these materials have a good application prospect. When 2M-DDF was employed as the guest molecules in an afterglow material, the afterglow time of 2M-DDF was 2.5 times that of TPD. Notably, the afterglow persisted for >10 s. The structure–activity relationship of these molecules was systematically described, providing a theoretical foundation for broadening their application in photophysical devices.

## 2. Results and Discussion

### 2.1. Fluorene-Based Amorphous Compounds with Good Solubility and High Thermal Stability Facilitate the Preparation of Flexible Films via Solution-Processable Materials Methods

**Figure 1:**
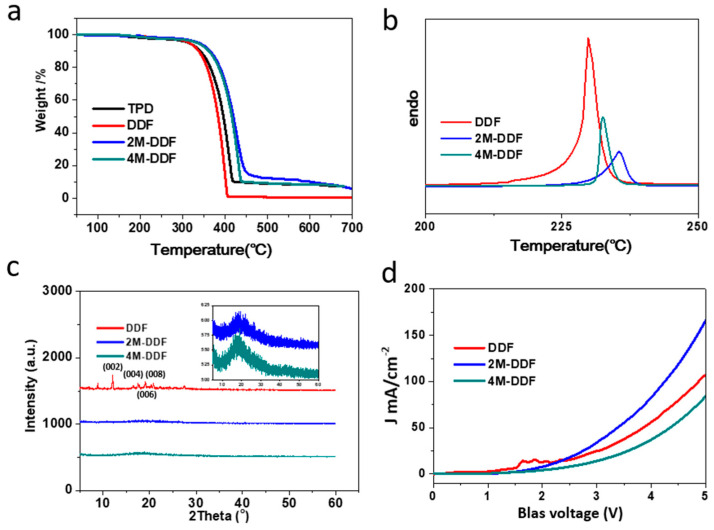
TGA (**a**), DSC (**b**), XRD (**c**), and SCLC (**d**) curves of DDF, 2M-DDF, and 4M-DDF. The space charge limited current (SCLC) of hole-only devices with the configuration of indium tin oxide (ITO)/poly(3,4-ethylenedioxythiophene)–polystyrene sulfonate (PEDOT:PSS)/DDF (red), 2M-DDF (blue), 4M-DDF (cyan)/Au.

The chemical structure and synthetic routes of DDF, 2M-DDF, and 4M-DDF are shown in Scheme S1 of the Supporting Information. Solubility is an important property of a compound and directly affects the performance of a device. Good solubility is a prerequisite for the preparation of high-performance devices [40,41]. Thermodynamic stability is critical to the performance of materials. The TGA and DSC properties of fluorene-based small molecules DDF, 2M-DDF, 4M-DDF were tested at a heating rate of 10 °C min^−1^ in a nitrogen atmosphere. From the TGA curves in Figure 1a, it can be seen that the temperatures corresponding to 5% mass loss of DDF, 2M-DDF, and 4M-DDF compounds are 319, 329, 327, and 348 °C, respectively, indicating good thermal stability. The DSC curves in Figure 1b show that the melting points of DDF, 2M-DDF, and 4M-DDF are 185, 230, 236, and 232 °C, respectively, indicating high melting temperatures and good thermal stability for the fluorene-based small molecules.

The synthesized compounds were easily crystallized during the spin-coating preparation of thin films. Grain boundaries that impede the carrier transport were easily formed. Introducing a methyl group at the end group of the molecule increased the steric hindrance, effectively inhibiting molecular crystallization as shown by the X-ray diffraction (XRD) patterns in Figure 1c. The XRD spectrum of DDF displays obvious crystalline peaks but those of 2M-DDF and 4M-DDF show diffuse low peaks (Figure 1c inset), confirming their amorphous nature. As hoped, the methyl group strongly inhibited the crystallization properties of the compound. A smooth, amorphous 2M-DDF film was prepared using the solution method [42]. The well-formed 2M-DDF film is a promising solution-processable HTM for photovoltaic devices such as OLEDs and organic photoconductors.

### 2.2. Superior Electrochemical Properties of Fluorene Compounds Facilitate Hole Injection and Hence the Carrier-Transport Efficiency

**Figure 2:**
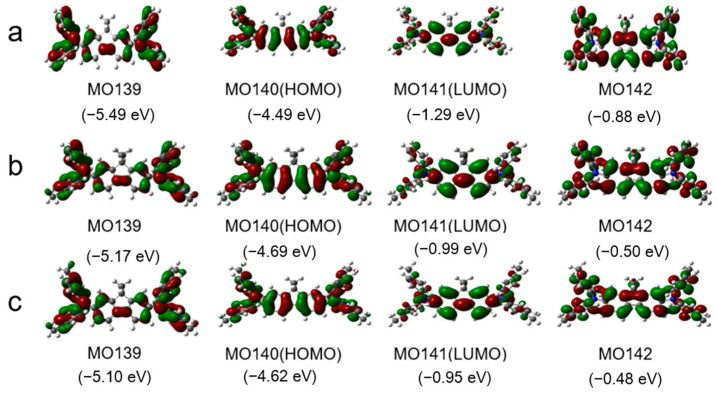
Some molecular orbitals and the corresponding energies of DDF (**a**), 2M-DDF (**b**), and 4M-DDF (**c**) using B3LYP/6-311G (d, p) in toluene. The grey, blue, and white spheres represent carbon, nitrogen, and hydrogen atoms, respectively.

**Figure 3:**
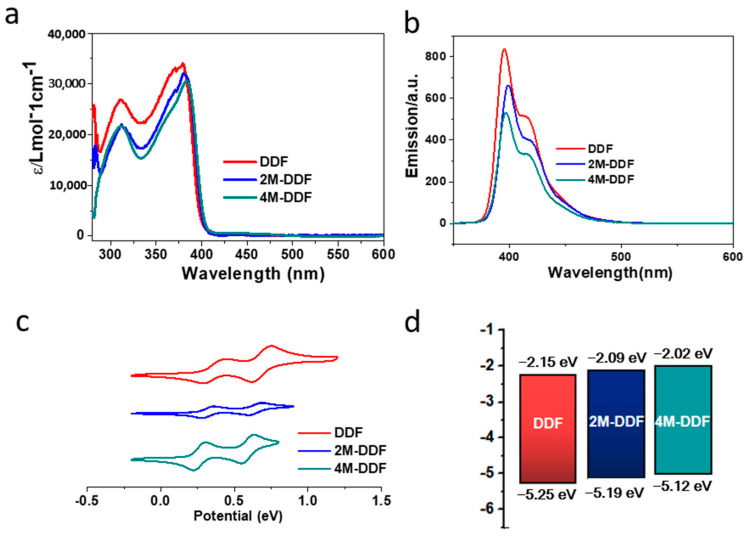
UV–vis absorption spectra (**a**) and fluorescence spectra (**b**) of DDF, 2M-DDF, and 4M-DDF in toluene (excitation wavelength: λ_max_ of absorption; concentration: 5 × 10^−6^ mol L^−1^). The UV–vis absorption and fluorescence were measured at room temperature. (**c**) Electrochemical curves of DDF, 2M-DDF, and 4M-DDF in dichloromethane vs. Ag/Ag^+^, with the concentration of 5 × 10^−3^ mol L^−1^ and orbital energy levels of those determined (**d**). The HOMO energy was deduced from the oxidation onset potential and calculated by the equation: E_HOMO_ = −E_onset_ − 4.93 (eV), E_LUMO_ = E_HOMO_ + Eg_op._ The optical band gap (Eg_op_) was calculated using Tauc’s relation in toluene [43,44].

Figure 2 shows the frontier orbitals of the arylamines calculated using density functional theory (DFT) at the B3LYP/6-31G(d) level. Introducing the fluorene group and adjusting the end groups largely affected the energy level of the compound. The HOMO energy levels of DDF, 2M-DDF, and 4M-DDF were −4.99, −4.69, and −4.62 eV, respectively. The LUMO energy levels of DDF, 2M-DDF, and 4M-DDF were −1.29, −0.99, and −0.95 eV, respectively, indicating that the energy level can be adjusted by changing the molecular structure. Figure 3a depicts the UV–vis absorption and fluorescence (FL) spectra of DDF, 2M-DDF, and 4M-DDF in toluene solution (5 × 10^−6^ M). The spectra of all compounds show a strong absorption peak around 380–400 nm assignable to n–π* transitions of the fluorene core [43]. From the onset of absorption, the optical band gap *E*_g_ was calculated as 3.10 eV for DDF, 2M-DDF, and 4M-DDF. The fluorescence maxima of the fluorene-based compounds appeared at 410 nm (Figure 3b). Figure 3c shows the electrochemical measurements (CV curves) of DDF, 2M-DDF, and 4M-DDF. From the onset potentials of oxidation, the HOMO energy levels of DDF, 2M-DDF, and 4M-DDF were estimated as −5.25, −5.19, and −5.12 eV, respectively. Note that all fluorine-based compounds exhibited shallower HOMO energies than TPD (−5.50 eV) [16,17]. The HOMO energies of 2M-DDF (−5.19 eV) and 4M-DDF (−5.12 eV) approached the work function energy of indium tin oxide (ITO) (−4.80 eV), implying smaller hole-injection barriers from ITO to 2M-DDF and 4M-DDF (0.39 and 0.32 eV, respectively) than from ITO to TPD [16,17]. The reduced injection barrier of holes is conducive to hole injection. From the HOMO energy levels and Eg_op_, the lowest unoccupied molecular orbital (LUMO) energy levels of DDF, 2M-DDF, and 4M-DDF were calculated as −2.15, −2.09, and −2.02 eV, respectively. The low LUMO energies of 2M-DDF and 4M-DDF indicate that these materials can prevent electron leakage from the emissive layer into the hole-transport layer. Judging from the energy levels, all compounds are suitable hole-transport materials for optoelectronic devices. 2M-DDF and 4M-DDF with higher HOMO energies are especially expected to improve the carrier-transport performance.

### 2.3. Superior Carrier-Transport Performance and High Energy Level Greatly Improve the Luminous Efficiency of OLED Devices

The electroluminescent efficiency of an HTM critically depends on the hole-injection/transporting capability of the material. To measure the hole mobilities of the HTMs, we fabricated hole-only devices with the configuration ITO/PEDOT:PSS/HTMs/Au (PEDOT:PSS = poly(3,4-ethylenedioxythiophene) polystyrene sulfonate). The hole mobilities of TPD, DDF, 2M-DDF, and 4M-DDF were measured using the space charge limited current method. The HTM layers were prepared by spin coating. The current density–voltage (*J–V*) characteristics of the single-carrier devices are shown in Figure 1d. All fluorine-based compounds generated much higher hole-current densities than TPD, owing to the hole-predominant transporting ability of triphenylamine. The hole mobilities of DDF, 2M-DDF, and 4M-DDF were 2.35 × 10^−4^, 4.65 × 10^−4^, and 1.55 × 10^−4^ cm^2^ V^−1^ s^−1^, respectively (Figure 1d), far exceeding that of TPD (1 × 10^−4^ cm^2^ V^−1^ s^−1^) [16,17]. The especially high mobility of 2M-DDF (five times that of TPD) can be explained partly by the good film-forming properties of the fluorene-based 2M-DDF molecule. The smaller the interface barrier, the more favorable is the carrier transport. Another contributing factor is the higher HOMO energy of 2M-DDF (−5.19 eV) compared to TPD (−5.50 eV) [16,17]. The close HOMO energy levels of 2M-DDF and ITO (−4.80 eV) reduce the injection barrier of holes, facilitating hole injection [13]. Owing to their superior transmission properties, 2M-DDF thin films fabricated via solution-engineering spin coating were chosen as the hole-transport material in OLED devices.

**Figure 4:**
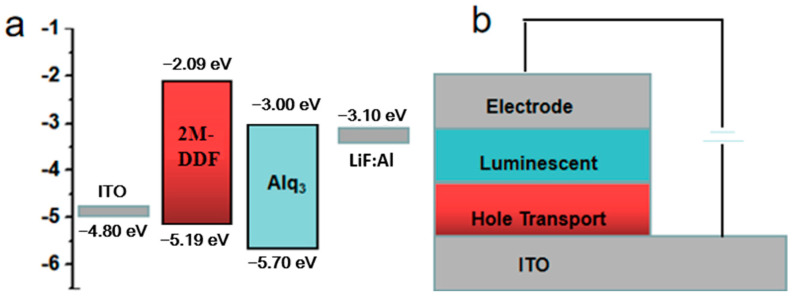
(**a**) Schematic energy level diagram of the fabricated OLED devices. (**b**) The device structure of OLEDs.

**Figure 5:**
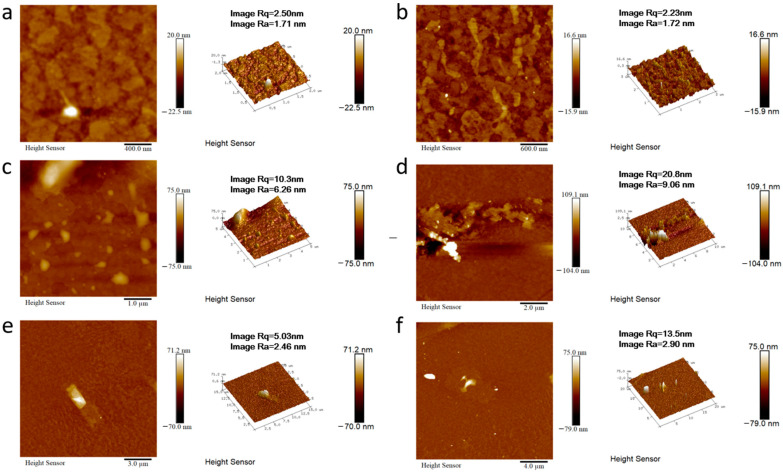
AFM height images of ITO/2M-DDF with different multiples ((**a**): 400 nm, (**b**): 600 nm, (**c**): 1 μm, (**d**): 2 μm, (**e**): 3 μm, (**f**): 4 μm).

In order to better expand the application field of this kind of materials, we take the materials of 2M-DDF with the highest carrier mobility as an example to explore its application in the field of OLED. 2M-DDF was inserted as the hole-transport layer in OLED devices (ITO/HTL/Alq3/LiF/Al). The energy levels in the materials and the device structures of the OLEDs are displayed in Figure 4a and Figure 4b, respectively. The relevant parameters of the fluorescent OLEDs are summarized in Table 1. With 2M-DDF as an HTM, the OLED device achieved a maximum *L* (*L*_max_) of 21,412 cd m^−2^ and a maximum *CE* (*CE*_max_) of 4.78 cd A^−1^, with a turn-on voltage of *V*_on_ = 3.8 V. It is widely believed that the film morphology of the functional layer is critical for the performance of multilayered optoelectronic devices. To further verify the film morphology of 2M-DDF as HTMs, we performed atomic force microscopy (AFM) measurements on the film samples of ITO/2M-DDF. As shown in Figure 5, tapping-mode AFM images of the ITO/2M-DDF show smooth surface morphology. The advantages of this material were better demonstrated by comparing it with the similarly structured compound TPD as a hole-transport layer device [45]. The *L*_max_ and *CE*_max_ of the TPD device were 4106 cd m^−2^ and 3.70 cd A^−1^, respectively, far below those of 2M-DDF, and the *V*_on_ increased to 4.3 V. The OLED device primarily utilizes evaporation for the hole-transport layer, with TPD being the chosen material. To better showcase the advantages of a solution-prepared hole-transport layer, we have selected poly(9-vinylcarbazole) (PVK) and poly[(9,9-dioctylfluorenyl-2,7-diyl)-alt-(4,4′-(N-(4-butylphenyl) (TFB) as contrast materials for performance comparison. The L_max_, CE_max_, and V_on_ of the PVK device were 2288.9 cd m^−2^, 1.10 cd A^−1^, and 3.4 V, respectively. It is due to the low HOMO energy level of PVK, which leads to a large hole injection barrier between ITO and PVK. In addition, PVK also has a low charge mobility of 2.5 × 10^−6^/V⋅s [12,15,46,47,48,49]. The *L*_max_, *CE*_max_ and *V*_on_ of the TFB device were 15,211 cd m^−2^, 2.90 cd A^−1^, and 2.4 V, respectively. The especially high luminous brightness of 2M-DDF (nearly five times that of TPD and nearly 1.3 times that of TFB) is further substantiated by the fact that this particular hole-transport material exhibits significant potential for application within the realm of OLED technology. Moreover, it provides a theoretical basis for how to design molecules with higher mobility.

**Figure 6:**
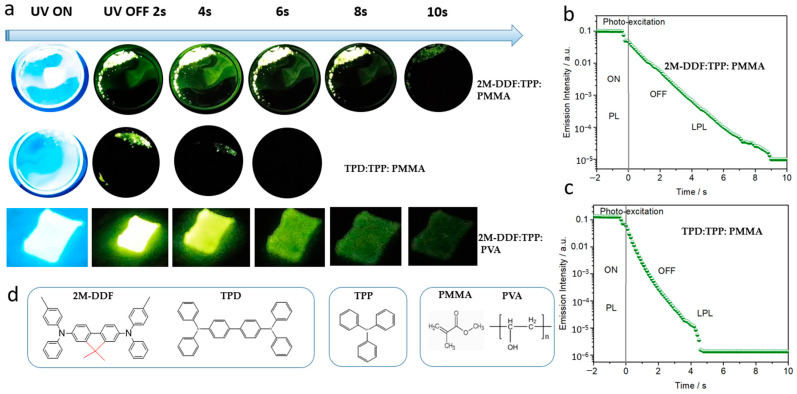
(**a**) LPL photographs under ambient conditions of 2M-DDF:TPP:PMMA, TPD:TPP:PMMA, and 2M-DDF:TPP:PVA (excitation: 365 nm). The semi-logarithmic plot of the emission decay profile of the 2M-DDF:TPP:PMMA (**b**) and TPD:TPP:PMMA (**c**) from −2 s to 10 s, which exhibited photoluminescence (PL) upon photo-excitation (from −2 s to 0 s) and LPL after the excitation turned off (excitation wavelength: 365 nm; excitation power: 10 mW; excitation time: 2 s; sample temperature: 300 K). (**d**) Chemical structure of 2M-DDF, TPD, TPP, PMMA, and PVA.

To better reflect the advantages of these kinds of effective carrier-transport materials based on 2M-DDF, we used 2M-DDF/TPD and triphenylphosphine (TPP) materials as guests and hosts and poly (methyl methacrylate (PMMA) polymers as rigid matrices to prepare rigid 2M-DDF:TPP:PMMA and TPD:TPP:PMMA. The 2M-DDF:TPP:PMMA afterglow materials were irradiated under a 365-nm ultraviolet lamp for 5 s and the light source was then removed. The 2M-DDF:TPP:PMMA emitted a green afterglow for 10 s (upper row of Figure 6a), which was clearly visible to the naked eye. TPD:TPP:PMMA also emitted a visible green afterglow, but its lifetime was only 4 s (lower row of Figure 6a). Figure 6b shows the semi-logarithmic plots of the emission decay profiles of 2M-DDF:TPP:PMMA and TPD:TPP:PMMA from −2 s to 10 s. Both 2M-DDF:TPP:PMMA and TPD:TPP:PMMA exhibit photoluminescent characteristics under light excitation (−2 to 0 s), and long afterglow characteristics after turning off the excitation light source (0–12 s). The long-persistent luminescence emissions of 2M-DDF:TPP:PMMA and TPD:TPP:PMMA visibly endured for approximately 10 s and 4 s, respectively, consistent with the afterglow times under naked eye observation. At present, there are still few afterglow materials with more than 10 s, and this research provides a basis for the development of materials with longer afterglow (as shown in Table 2) [50,51,52,53]. The comparison with TPD as a hole-transport material with high carrier mobility reveals that 2M-DDF is more suitable for the preparation of afterglow materials.

The material prepared on the PMMA substrate is a rigid film suitable for applications requiring greater hardness. We also prepared polymer films on substrates of polyvinyl alcohol (PVA) (2M-DDF:TPP:PVA and TPD:TPP:PVA) and polyethylene glycol (PEG) (2M-DDF:TPP:PEG and TPD:TPP:PEG) (Figure 6a). The films were simply and quickly prepared by heating the compounds. As expected, the PVA substrate realized a flexible film with similar afterglow times as PMMA (9 s for 2M-DDF:TPP:PVA; see upper row of Figure 6a). Clearly, the polymer-substrate characteristics strongly influence the afterglow time, implying a wide application range of the 2M-DDF material.

**Figure 7:**
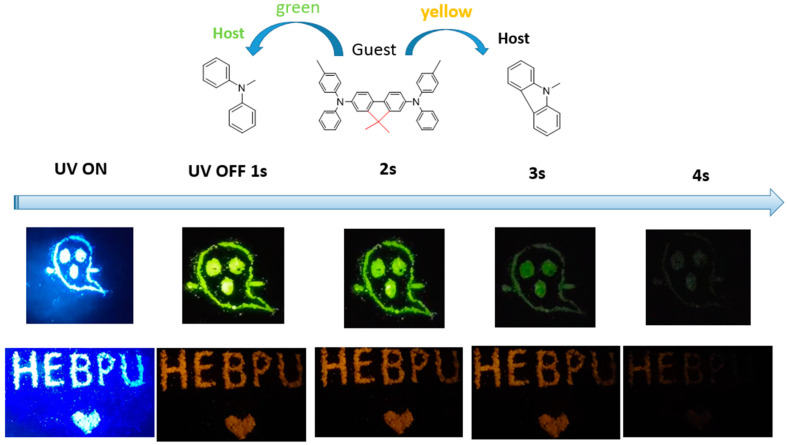
The LPL photograph under ambient conditions of 2M-DDF:DPA (**up**) and 2M-DDF:CA (**down**) (excitation: 365 nm).

To further expand the application field of the proposed materials, we doped crystal materials with different hosts of different sizes, preparing multi-color tunable afterglow materials in a rigid crystal-mode environment. When diphenylamine (DPA) and CA host materials were doped with 2M-DDF guest molecules (forming doped crystalline 2M-DDF: DPA), we were delighted to observe a deep yellow afterglow for 3 s and a deep green afterglow for 3.4 s under 365 nm ultraviolet excitation (see Figure 7). Such multi-color tunable afterglow materials can potentially be prepared as anti-counterfeiting materials with high encryption degrees.

In order to better expand the application of materials, we replaced the spiro-OMeTAD hole-transport layer with 2M-DDF in the fabrication of perovskite solar cells. Unfortunately, the efficiency of the replaced spiro-OMeTAD hole-transport layer with 2M-DDF in the fabrication of perovskite solar cells is lower 2M-DDF(1)~2M-DDF(3) (as shown in Table S1). Analysis of the test data shows that the relatively high FF% of the replaced spiro-OMeTAD hole-transport layer with 2M-DDF suggests suitable mobility of 2M-DDF (as shown Table S1); however, the unsuitable energy levels and the suboptimal PVK/HTM interface increase charge recombination, thereby reducing device efficiency. We attempted to incorporate 2M-DDF as an intermediate layer between the perovskite and hole-transport layer. The corresponding test results are presented in rows 2M-DDF(4)~2M-DDF(6) of Table S1. However, the results remained suboptimal, with considerably low photoelectric conversion efficiency. This limitation is primarily attributed to the mismatch between the perovskite and 2M-DDF layers. As reported in the literature, both high hole mobility and resistance to morphological perturbations are critical for effective HTMs [54,55,56].

## 3. Conclusions

This study analyzed the properties of three newly synthesized solution-processable HTMs (DDF, 2M-DDF, and 4M-DDF). The energy level, solubility, crystallinity, and molecular steric configuration could be simultaneously tuned by changing the number of terminal methyl groups. Among the synthesized compounds, 2M-DDF exhibited the highest hole mobility (4.35 × 10^−4^ cm^2^ V^−1^ s^−1^) compared to DDF (2.35 × 10^−4^) and 4M-DDF (1.55 × 10^−4^ cm^2^ V^−1^ s^−1^). Consequently, devices fabricated with 2M-DDF as HTMs with an Alq_3_ emitter delivered a maximum CE of 4.78 cd/A and a maximum *L* (*L*_max_) of 21,412 cd m^−2^ with a turn-on voltage of *V*_on_ = 3.8 V. The luminous efficiency of 2M-DDF was approximately five times that of TPD and 1.3 times that of TFB. The proposed strategy can simply design solution-processable HTMs for efficient OLEDs. Moreover, 2M-DDF and TPD can be incorporated as guest molecules in afterglow materials. The afterglow time of 2M-DDF was 2.5 times that of TPD and endured for a surprisingly long time (10 s). This study provides a theoretical basis for developing high-performance organic photoelectric devices and long-afterglow materials. The afterglow time and brightness of green afterglow and deep yellow afterglow materials have been significantly enhanced; however, further advancements are required in the development of other color afterglow materials. For example, one of the common applications of organic RTP materials is biological imaging, where the beneficial emission wavelength is in the near-infrared region. However, there are only a few related studies at present, so that finding a suitable host matrix is an important way to activate near-infrared RTP emission. On the other hand, in future studies, a new material with enhanced hole mobility and a suitable energy level should be developed to replace the conventional spiro-OMeTAD in perovskite solar cells. By refining the structure of the compound to optimize its energy level, we can better support efficient hole transport in perovskite applications.

## Figures and Tables

**Table 1:** EL performance of the fabricated OLED devices.

Compound	V_on_ ^a^ (V)	CE ^b^ (cd/A)	PE ^c^ (lm/W)	Lmax ^d^ (cd/cm^−2^)
TPD [45]	4.3	3.70	15	4106
TFB only [12]	2.4	2.90		15,211
PVK only [12]	3.4	1.10		2288.9
TFB/PVK [13]	7.4	5.35		10,520
2M-DDF [46]	3.8	4.78	16	21,412

^a^ Turn-on voltage (V), recorded at luminance of 1 cd/m^2^. ^b^ Maximum current efficiency. ^c^ Maximum power efficiency. ^d^ Maximum luminance.

**Table 2:** A summary of properties of organic LPL materials.

No.	Host	Guest	Processing Technique	Time	Afterglow Color	Ref.
1	9H-carbazole	9H-carbazole	crystallization	2 s	Orange	[50]
2	2,8-bis(diphenylphosphoryl)dibenzo[b,d]	2,7-di-(N,N-diphenylamino) 9,9-dimethyl-9H-fluorene	crystallization	6 s	Blue	[51]
3	PMMA:TPP	(R)-1,1′-Bi-2 naphthylamine	heat	10 s	Blue	[52]
4	triphenylphosphine	N,N′-diphenyl-dihydrodibenzo [a,c]phenazine derivatives	crystallization	3 s	Blue	[53]

## Data Availability

The original contributions presented in the study are included in the article/Supplementary Material, further inquiries can be directed to the corresponding author.

## Supplementary Materials

The following supporting information can be downloaded at: https://www.mdpi.com/article/10.3390/ma17225417/s1, Scheme S1. Synthetic routes and chemical structure of 1 and 2a–c (1) CH_3_I, t-BuOK, THF, rf, 12 h, Ar; (2) K_2_CO_3_, Cu, NaHSO_3_, Toluene, 210 °C, 10 h; The star to showcase the binding site (N-group) to the fluorene. Table S1. Summary of the photovoltaic performance of PSC devices.
