# Supplementary material for: Hole-Transporting Materials Based on a Fluorene Unit for Efficient Optoelectronic Devices
Source: Materials (Basel). 2024 Nov 6;17(22):5417. doi: 10.3390/ma17225417 (PMC11595885; doi:10.3390/ma17225417)
Supplement: Supplementary file 1 [file materials-17-05417-s001.zip › materials-3261073-supplementary.pdf]

Hole-Transporting Materials Based on a Fluorene Unit for Efficient Optoelectronic Devices

Maoli Man [1], Mingming Zhao [2] and Yunfei Lyu [1,*]

[1] Hebei Petroleum University of Technology, Chengde 067000, China;

[2] School of Chemical Engineering and Technology, Tianjin University, Tianjin 300354, China;

[*]E-mail: **lvyunfei4321@163.com**

Corresponding Author

Yunfei Lyu , E-mail: **lvyunfei4321@163.com**

Table of Contents

Section S1 Characterization data 2

Section S2 Synthesis of compounds 4

Section S3 References 9

Section S1 Characterization data

^{1}HNMR and ^{13}CNMR: ^{1}HNMR and ^{13}CNMR spectra were obtained on a Varian INOVA-500MHZ superconducting spectrometer.

ESI and HR-ESI: The ESI mass and HR-ESI mass spectra were obtained on a Thermo Fisher LCQ Deca XP MAX mass spectrometer.

MALDI-TOF and HR-MALDI-TOF: MALDI-TOF mass and HR-MALDI-TOF mass spectra were obtained on a microOTOF-Q II mass spectrometer respectively.

UV-Vis: UV-Vis Spectroscopy in solution. We obtained the absorption spectra using the Thermo Spectronic, Helios Gamma spectrometer. Quartz cells has a path length of 1 cm.

TGA. The thermal stability of the samples was determined by measuring the weight loss while heating at a rate of 10 ºC min^{-1} from 30 ºC to 700 ºC under inert nitrogen atmosphere.

DSC. The samples of DDF and PPT were heated at a rate of 10 ºC min^{-1} from 30 ºC to 350 ºC under inert nitrogen atmosphere.

CV. The electrochemical properties were measured using a BAS 100W electrochemical analyzer utilizing the three-electrode configuration with a glassy carbon electrode as the working electrode, $Ag/AgNO_3$ electrode as the reference electrode, and platinum as the auxiliary electrode. The analyzer was calibrated using a ferrocene/ferrocenium redox couple as the external standard prior to the measurements. The scan rate was set to 30 mV/s. Dichloromethane containing 0.1 mol•L^{-1} tetra-butylammoniumhexafluorophosphate ($TBAPF_6$) was employed as the medium for the cyclic voltammetric determination. The compound concentration was 5×10^{-3} mol•L^{-1}.

SCLC: The hole-only devices were fabricated with configuration of ITO/PEDOT:PSS/blend/Au. Subsequently, the blend was spin-coated on it under the same condition as preparation of the optimal solar cell. The Au layer was thermally deposited on the top of the blend in vacuum. The Au layer was deposited under a low speed to avoid the penetration of Au atoms into the active layer. The current densitye/voltage curves of the devices were recorded with a Keithley 2400 source.

OLED fabrication (dry)： The devices of 2M-DDF with the following structures were fabricated in the experiments as reported in the literature[1-3]: ITO/Au(5 nm)/SAM/2M-DDF(50 nm)/Alq3(40 nm)/TPBI(15 nm)/LiF(1 nm)/Al(100 nm).

OLED fabrication (solution spin coating): Devices with sandwich ITO/HTL/Alq3/LiF/Al structures were prepared. ITO glass substrates were sequentially sonicly rinsed with isopropanol, acetone, and deionized water, and treated with oxygen plasma for 5 min. The cross-linked films were prepared by spinning toluene-dissolved 2M-

DDF solution (70 μL, 10 mg/mL) at a rate of 4 000 r/min, spinning for 30 s, and then annealing at 100 °C for 30 min to remove residual solvent. Alq3 film at a pressure of about 6 × 10^{-4} Pa at 0. A rate of 1 nm/s was deposited on the substrate, after which LiF and Al were sequentially deposited on the substrate. The EL spectrum, device brightness and CIE coordinates were measured with Konica minolta CS-2000 (Konica Minolta, Japan). Current-voltage characteristics were recorded using a Keithley 2400 digital source meter (Keithley, United States). All measurements were taken at room temperature.

Preparation of trace doped crystal 2M-DDF: DPA: At room temperature, 5 mL of anhydrous ethanol was added to 1 mol % MDPA: DDF-O mixed powder (0.50 g), and the solution was ultrasonically dispersed using an ultrasonic cleaner to dissolve and uniformly mix the donor and acceptor materials. Then 50 mL of deionized water was slowly added to the solution in the presence of air to produce a white precipitate. The suspension was left at room temperature for 6 hours to allow the sediment to settle completely. The resulting solid was collected by filtration, yielding a white crystalline product.

The preparation of trace-doped crystals 2M-DDF:CA is similar to the preparation process of trace-doped crystals 2M-DDF:DPA, which only needs to replace the CA in the raw material with DPA.

Preparation of trace doped crystal 2M-DDF：PMMA: 2M-DDF, triphenylphosphine and PMMA were measured at a mass ratio of 1:30:100 into a 10 mL vial. The vial was placed on a constant temperature heating table, heated and melted at 160°C for 20 minutes, and cooled for later use.

Preparation of trace doped crystal 2M-DDF：PEG: 2M-DDF triphenylphosphine and PEG were measured at a mass ratio of 1:30:100 into a 10 mL vial. The vial was placed on a constant temperature heating table, heated and melted at 180°C for 15 minutes, and cooled for later use.

Preparation of trace doped crystal 2M-DDF：PVA: 2M-DDF triphenylphosphine and PVA were measured at a mass ratio of 1:30:100 into a 10 mL vial. The vial was placed on a constant temperature heating table, heated and melted at 190°C for 25 minutes, and cooled for later use.

Ultra-thin gold films of 5 nm thickness were deposited onto freshly cleaned ITO substrates. The OLED devices were fabricated using a thermal evaporator on top of ITO. A typical deposition procedure was as follows: at a pressure below 10^{-5} Torr, a 50-nm-thick

layer of 2M-DDF was first deposited, followed by 40 nm of Alq_3 and 15 nm of TPBI at a deposition rate of 2–3 Å/ s . Next, a 1-nm-thick LiF layer was deposited. Finally, a 100-nm-thick Al cathode was deposited through a shadow mask. This metallic layer was patterned via a shadow mask to produce four devices, each with an area of 0.06 cm^2. The current-voltage forward luminance characteristics of each device were measured using a ST-900 M luminance meter and a Keithley 2400 programmable voltage current source (Tektronix, USA).

Device preparation process of ITO/SnO_2/PVK/PEAI/2M-DDF/Ag (2M-DDF(1)~2M-DDF(3)).
The ITO glass substrates were ultrasonically treated with detergent, deionized water, acetone, and ethanol for 20 min each. After cleaning, the substrates were dried with dry nitrogen gas and treated with ultraviolet–ozone for 30 min. A diluted SnO_2 colloid solution (3.75 wt.%) was then spin-coated onto the substrates at 4000 rpm for 30 s, followed by annealing at 150°C for 30 min to form a compact SnO_2 electron transport layer. After cooling to room temperature, the substrate subjected to ultraviolet–ozone treatment for 10 min. A 1.65-M perovskite precursor solution was prepared using the one-step "anti-solvent" method. In particular, 705.3 mg of PbI_2, 240.8 mg of FAI, 33.8 mg of MACl, 17.0 mg of MABr, and 56.0 mg of $PbBr_2$ were dissolved in 1 mL of DMF/DMSO (volume ratio = 8:1) with a chemical formula of $(FA_{0.9}MA_{0.1}PbI_3)_{0.9}(MAPbBr_3)_{0.1}$. This solution was spin-coated on top of ETL at 1000 rpm for 10 s and then at 5000 rpm for 20 s. During the last 10 s, absolute ether was dropped onto the perovskite film as an anti-solvent. The sample was then annealed at 100 °C for 60 min (10%–20% humidity). Further processing was performed in an N_2-filled glovebox. Next, a PEAI solution (3 mg mL^{-1} in IPA) was spin-coated onto the perovskite surface at 5000 rpm for 30 s without further processing. A 70-μL 2M-DDF compound solution (10 mg mL^{-1} in toluene) was then spin-coated on the perovskite layer at 4000 rpm for 30 s. For the control device, a 40-μL spiro-OMeTAD solution, prepared by dissolving 90 mg of spiro-OMeTAD, 29 μL *t*-BP, and 20 μL Li-TFSI solution (540 mg Li-TFSI in 1 mL ACN) in 1 mL CB, was spin-coated on the perovskite layer at 3000 rpm for 30 s. Finally, an 80-nm Ag electrode was deposited on top of the HTL via thermal evaporation.

Device preparation process of ITO/SnO_2/PVK/PEAI/2M-DDF and spiro-OMeTAD /Ag (2M-DDF/ spiro-OMeTAD (4)~2M-DDF/ spiro-OMeTAD (6)).
The other steps are the same as Device preparation process of ITO/SnO_2/PVK/PEAI/2M-DDF/Ag (2M-DDF(1)~2M-DDF(3)).
Preparation 2M-DDF semiconductor layer

For 2M-DDF semiconductor layer preparation, the 2M-DDF (3.8 mg)/$(MTPA)_2Ab$ to dissolve in a 50 μL mixture of toluene and isopropanol (4:1) , mix well. Then spin-coated onto the perovskite surface (40 μL) at a spin rate of 4000 r / min and a rotation time of 30 s. Without any further processing.

Preparation of modified Spiro-OMeTAD hole transport layer

A solution of spiro-OMeTAD as a hole transporting material (HTM) (73.5 mg) was mixed with 17.5 μL bis(trifluoromethylsulfonyl) imide lithium salt (Li-TFSI) solution (520 mg acetonitrile), 8 μL tris(2-(1Hpyrazol- 1-yl)-4-tert-butylpyridine)-cobalt(III)tris-(bis(trifluoromethylsulfonyl)imide) solution (300 mg/mL acetonitrile), and 28.5 μL 4-tert-butylpyridine (t-BP) in chlorobenzene. The resultant solution was deposited on top of the CS semiconductor layer film by spin coating at 4000 rpm for 20 s. Oxidation in a dry box overnight.

Computations. Geometry optimizations of the molecules were carried out using the three-parameter exchange functional of Becke and correlation functional of Lee, Yang and Parr (B3LYP)[4-6] with the 6-311G(d,p) basis set in toluene without any symmetry constraints. The polarized continuum model (PCM) framework[7] was used to describe the solvent effect. The SCF convergence was 10^{-8} a.u. while the gradient and energy convergence was 10^{-4} a.u. and 10^{-5}a.u., respectively. All the calculations were performed using *Gaussian09* package[8]. In order to confirm the optimized geometry as a global minimum, frequency calculations at the same level of theory were performed. The calculations were performed following the same procedures as our previous work[9,10].

Section S2 Synthesis of compounds

Materials.

Potassium carbonate (K_2CO_3), copper(Cu), Sodium Hydrogen Sulfite($NaHSO_3$), 2,7-diiodo-9,9-dimethyl-9H-fluorene (AR, 98%) were bought from Sinopharm Chemical Reagent Co. Ltd. Other reagents were commercially available and used without further purification.

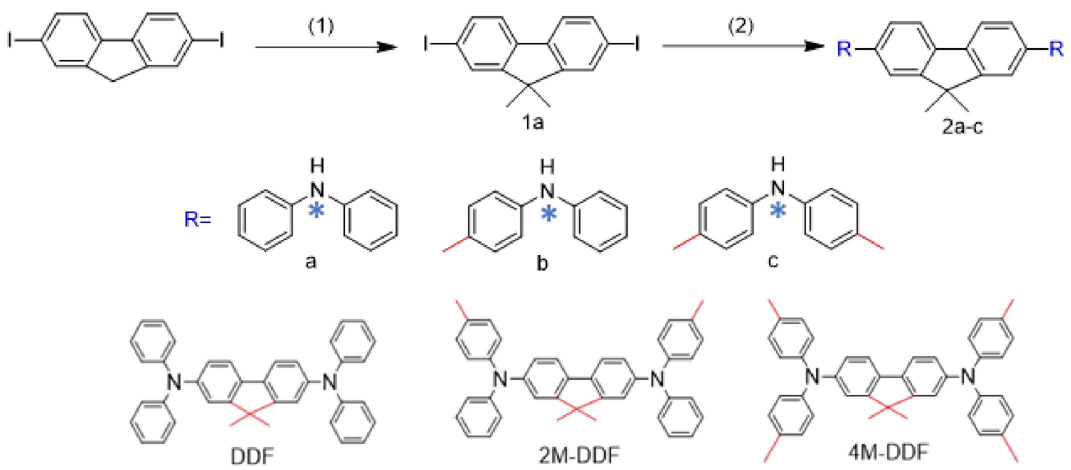

Scheme S1. Synthetic routes and chemical structure of 1 and 2a-c (1) CH_3I, t-BuOK, THF, rf, 12h, Ar; (2) K_2CO_3, Cu, $NaHSO_3$, Toluene, 210°C, 10h;The star to showcase the binding site (N-group) to the fluorene.

Synthesis of compound 1a

2,7-diiodo-fluorene (0.50 g, 1.196 mmol) were dissolved in THF (10 mL) at an ice bath in nitrogen atmosphere and the suspension was stirred 10 min. Then potassium tert-butoxide (0.40 g, 3.588 mmol) was added into the suspension. Afterwards the suspension was stirred for 30 min. Then CH_3I (0.22 mL, 3.580 mmol) was added dropwise into the suspension. The suspension was stirred at room temperature for 12 h. After filtration, the solid was washed with absolute dichloromethane. Then the organic phases were condensed by rotary evaporation. The product was further purified by column chromatography on silica gel using cyclohexane: dichloromethane (2:1= v:v) as the eluent.

A yellow solid (0.46 g, 86%)

^{1}H NMR($CDCl_3$, 500 MHz) δ:7.76 (d, *J* = 1.6 Hz, 2H), 7.68 (dd, *J* = 8.0 Hz, 1.6 Hz, 2H),

7.45 (d, *J* = 8.0 Hz, 2H), 1.47 (s, 6H); ^{13}C NMR ($CDCl_3$, 101 MHz) δ:154.15, 136.81, 135.15, 131.01, 120.78, 92.10, 46.13, 25.80; ESI-HRMS (m/z): $[M+H]^+$Calcd for $C_{15}H_{12}I_2$, 445.902 8; found, 445.914 0.

Synthesis of compound 2

2,7-diiodo-9,9-dimethyl-9H-fluorene (5.0g, 11.21mmol), diphenylamine (4.61g, 27.28mmol), $NaHSO_3$(0.055g, 0.53 mmol), Cu(0.26 g, 4.0 mmol) and Na_2CO_3(2.07g, 15.0 mmol) were dissolved in tolurer (5 mL) at room temperature. Afterwards, the suspension was refluxed at 210℃ for 8 h. After filtration, the solid was washed with absolute dichloromethane. Then the organic phases were condensed by rotary evaporation. The product was further purified by column chromatography on silica gel using cyclohexane: dichloromethane (2:1= v:v) as the eluent.

9,9-dimethyl-N2,N2,N7,N7-tetraphenyl-9H-fluorene-2,7-diamine (2a)

A pale white solid (4.85 g, 82%).

^{1}H NMR ($CDCl_3$, 500 MHz) *δ*:7.51 (d, *J* = 8 Hz, 2H), 7.28 (t, *J* = 8 Hz, 4H), 7.26 (s, 2H), 7.21 (d, *J* = 7.5 Hz, 2H) , 7.14 (d, *J* = 8 Hz, 6H), 7.10 (t, *J* =7.5 Hz, 4H), 7.02 (t, *J* =7.5 Hz, 6H), 1.36 (s, 6H)； ^{13}C NMR ($CDCl_3$, 101 MHz) *δ*:155.28, 146.81, 134.42, 129.45, 124.19, 122.75, 121.55, 120.17, 119.07, 118.07, 47.03, 27.49, ESI-HRMS (m/z): $[M]^+$ Calcd for $C_{39}H_{32}N_2^+$, 528.2560; found, 528.2560.

9,9-dimethyl-N2,N2,N7,N7-tetraphenyl-9H-fluorene-2,7-diamine (2b)

A pale white solid (5.31 g, 85%).

^{1}H NMR ($CDCl_3$, 400 MHz) *δ*:7.49 (d, *J* = 8.2 Hz, 2H), 7.25 (t, *J* = 7.7 Hz, 4H), 7.16 (d, *J* = 1.9 Hz, 2H), 7.14～7.04 (m, 12H), 7.01 (d, *J* = 8.2 Hz, 4H), 2.36 (s, 6H), 1.46 (s, 6H)； ^{13}C NMR ($CDCl_3$, 101 MHz) *δ*:153.85, 145.67,143.55, 132.70, 131.42, 128.83, 128.03, 123.58, 122.32, 122.02, 121.11, 120.94, 119.22, 118.81, 117.28, 45.71, 25.98, 19.80.; ESI-HRMS (m/z): $[M]^+$ Calcd for $C_{41}H_{36}N_2^+$, 556.2873; found, 556.2873.

9,9-dimethyl-N2,N2,N7,N7-tetraphenyl-9H-fluorene-2,7-diamine (2c)

A pale white solid (5.43 g, 83 %).

^{1}H NMR ($CDCl_3$, 500 MHz) *δ*:7.44 (d, *J* = 8 Hz, 2H), 7.14-7.04 (m, 12H), 7.03 (d, *J* = 8 Hz, 8H), 6.94 (d, *J* = 8.5 Hz, 2H), 2.34 (s, 12H), 1.34 (s, 6H)； ^{13}C NMR ($CDCl_3$, 101 MHz) *δ*:155.03, 147.11,132.24, 130.01, 124.39, 122.75, 121.63, 119.92, 118.01, 53.47, 27.18, 21.05; ESI-HRMS (m/z): $[M]^+$ Calcd for $C_{43}H_{40}N_2^+$, 584.3191; found, 584.3189.

Table S1 Summary of the photovoltaic performance of PSC devices.

One step forward method		PCE%	J_{SC}(mA/cm^{-2})	Voc(V)	FF%
HTMs: 2M-DDF (1)	forward	0.058	5.36	0.0414	26.04
	reverse	0.086	5.79	0.0620	24.32
HTMs: 2M-DDF (2)	forward	0.352	10.73	0.108	30.33
	reverse	0.061	8.56	0.031	23.26
HTMs: 2M-DDF (3)	forward	0.264	10.89	0.0815	29.79
	reverse	0	-	-	-
HTMs: 2M-DDF/ spiro-OMeTAD (4)	forward	0.706	10.97	0.405	15.89
	reverse	1.034	12.50	0.321	25.79
HTMs: 2M-DDF/ spiro-OMeTAD (5)	forward	1.431	16.46	0.313	27.78
	reverse	1.655	16.48	0.307	32.72
HTMs: 2M-DDF/ spiro-OMeTAD (6)	forward	0.959	14.65	0.163	40.05
	reverse	0.228	13.09	0.065	26.62
No HTMs	forward	0	-	-	-
	reverse	0	-	-	-
HTMs: spiro-OMeTAD	forward	3.527	18.24	1.051	18.40
	reverse	3.700	18.26	1.055	19.19

Section S3 References

[1] C. C. Wu, C. I. Wu, J. C. Sturm, and A. Kahn, Surface modification of indium tin oxide by plasma treatment: An effective method to improve the efficiency, brightness, and reliability of organic light emitting devices. Appl. Phys. Lett. 1997, 70, 1348.

[2] Z. Shang, D. Liu, T. Wang, X. Yu, B. Li, W. Li, W. Hu, X. Zhou. Enhanced Hole-Injection Property in an OLED with a Self-assembled Monolayer of Hole-Transporting TPD on Thin Au as the Anode.Transactions of Tianjin University 2018, 24:580-586.

[3] S. Wu, H. Liua, W. Sun, X. Li, S. Wang. Regulation of peripheral tert-butyl position: Approaching efficient blue OLEDs based on solution-processable hole-transporting materials.Organic Electronics 2019, 71, 85-92.

[4] Becke, A. D. Density-functional exchange-energy approximation with correct asymptotic behavior. *Phys. Rev. A* **38**, 3098-3100 (1998).

[5] Becke, A. D. Density-functional thermochemistry. III. The role of exact exchange. *J. Chem. Phys.* **98**, 5648-5652 (1993).

[6] Lee, C. Yang, W. & Parr, R. G. Development of the colle-salvetti correlation-energy formula into a functional of the electron density. *Phys. Rev. B* **37**, 785-789 (1998).

[7] Cancès, E. Mennucci, B. & Tomasi, J. A new integral equation formalism for the polarizable continuum model: theoretical background and applications to isotropic and anisotropic dielectrics. *J. Chem. Phys.* **107**, 3032-3041 (1997).

[8] Frisch, M. J. Trucks, G. W. Schlegel, H. B. Scuseria, G. E. Robb, M. A. & Cheeseman, J. R. Gaussian 09, Revision D.01. Wallingford CT: Gaussian Inc. (2013).

[9] Wang, T. Y. Weerasinghe, K. C. Liu, D. Z. Li,W. Yan, X. L. Zhou X. Q. & Wang, L .C. Ambipolar organic semiconductors with cascades of energy levels for generating long-lived charge separated states: a donor-acceptor1-acceptor2 architectural triarylamine dye. *J. Mater. Chem. C* **2**, 5466-5470 (2014).

[10] Wang, T. Y. Weerasinghe, K. C. Sun, H. Y. Hu, X. X. Lu, T. Liu, D. Z. Hu, W. P. Li, W. Zhou, X. Q. & Wang, L. C. Effect of triplet state on the lifetime of charge separation in ambipolar D-A_1-A_2 organic semiconductors. *J. Phys. Chem. C* **120**, 11338-11349 (2016).
